# Clinicopathological characteristics and genomic profiling in patients with transformed lymphoma: a monocentric retrospective study

**DOI:** 10.1080/07853890.2024.2419556

**Published:** 2024-10-26

**Authors:** Xia Zhao, Haiyan Bian, Fengyun Hao, Shihong Shao, Chuanhong Wu, Qian Zhang, Mingxuan Wu, Zhiqiang Li, Chengwen Gao

**Affiliations:** a Department of Hematology, The Affiliated Hospital of Qingdao University, Qingdao, Shangdong, China; b Department of Pathology, The Affiliated Hospital of Qingdao University, Qingdao, Shangdong, China; c Laboratory of Medical Biology, Medical Research Center, The Affiliated Hospital of Qingdao University and The Biomedical Sciences Institute of Qingdao University (Qingdao Branch of SJTU Bio-X Institutes), Qingdao, Shangdong, China

**Keywords:** Transformed lymphoma, histological transformation, marginal zone lymphoma, clinical features, whole-exome sequencing

## Abstract

**Background:**

Transformed lymphoma occurs when indolent lymphoma transforms into more aggressive lymphoma usually associated with poor prognosis.

**Methods:**

In this study, we analysed the immunophenotypes, prognostic factors and outcomes of 35 patients with transformed lymphoma from among 306 marginal zone lymphoma (MZL), 544 follicular lymphoma (FL) and 871 chronic lymphocytic leukaemia/small lymphocytic lymphoma (CLL/SLL) cases. In addition, we performed whole-exome sequencing study of seven transformed MZL (tMZL) cases.

**Results:**

Our results demonstrate that the median time from indolent lymphoma diagnosis to transformed DLBCL was 35 months (range, 14–53 months). The 5-year overall survival (OS) and progression-free survival (PFS) rates after histological transformation (HT) were 50% and 26%, respectively. Kaplan–Meier survival analysis revealed that asynchronous HT and transformed CLL/SLL (tCLL/SLL) were significant adverse prognostic factors for OS after DLBCL HT. We identified mutations involvement in chromatin remodelling (*CREBBP* and *EP300*) and regulators of NF-κB signalling (*TNFAIP3, BCL10, MYD88, CD79B* and *CARD11*) were affected in tMZL.

**Conclusion:**

Whole-exome sequencing and copy-number analysis revealed that tMZL derives from the divergent evolution of an ancestral common progenitor clone (CPC). Collectively, this study provides clinicopathological characteristics of three common types of transformed lymphomas and the genetic profile of tMZL with diagnostic and therapeutic implications.

## Introduction

Transformation in lymphoma refers to the process by which a slow-growing (low grade) lymphoma evolves into a faster-growing (high grade) form, necessitating a different approach to treatment due to its increased aggressiveness. If histological transformation (HT) occurs, it typically leads to a rapidly advancing disease course and challenges in lymphoma management, potentially resulting in patient mortality. The most common subtype following transformation is diffuse large B-cell lymphoma (DLBCL) [1–4]. Any low-grade B-cell lymphoma can transform into DLBCL, including marginal zone lymphoma (MZL), follicular lymphoma (FL), and chronic lymphocytic leukaemia/small lymphocytic lymphoma (CLL/SLL).

Risk of HT associated with extranodal sites >1, performance status >1, B symptoms and elevated lactate dehydrogenase levels at initial diagnosis according to The National LymphoCare Study group report [5]. The rate of HT to DLBCL is approximately 2%–3% per year for FL [6], while the risk of HT is 0.3–1% per year after diagnosis for MZL lymphomas [7, 8]. The transformation rate of CLL/SLL reported in the literature approximately ranges from 2% to 10% [9, 10]. In recent years, an increasing number of genetic changes driving the transformation process have been discovered and exploited. However, in some patients with transformed lymphoma, the need for new therapies remains unmet, as many genes are focused on pathways that are not currently targetable, such as *MYC*, *CDKN2A* or deletion of *TP53* [1, 4].

Relevant studies have shown that the 5-year survival rate after the initial diagnosis in patients with HT is significantly lower than that in patients without HT [5, 7]. The 5-year survival rates for different types of transformed lymphomas are variable [1]. However, the prognostic factors and histopathological characteristics of different types of transformed lymphomas have not been sufficiently described.

In-depth characterization of the underlying genetic events leading from indolent lymphoma to transformed lymphoma will serve as a guide for the development of effective targeted therapies [11]. Currently, several studies have evaluated the transformed FL (tFL) genetic profile by high-throughput sequencing [11–14]. However, genomic studies on other types of transformed lymphomas are mostly case reports or small-scale studies. To the best of our knowledge, there have been no genomic studies of transformed MZL (tMZL) patients in Asian populations.

This study focuses on three common types of transformed lymphomas: tFL, tMZL, and [3] transformed CLL/SLL (tCLL/SLL). We provide a comprehensive review of the clinical and histopathological features of these three transformed lymphomas and performed the whole-exome sequencing study of tMZL. This study aimed to (i) assess the clinical features, prognostic factors, and histopathological characteristics of the three common types of transformed lymphomas, (ii) identify the molecular characteristics of tMZL.

## Materials and methods

### Patient selection

The study group comprised 35 patients with transformed lymphoma from among 544 FL, 306 MZL, and 871 CLL/SLL patients. The criteria of HT as follows: characterized by a diffuse structural arrangement and a minimum of 20% large lymphoma cells. In complex cases, a Ki-67 index of 50% or higher serves as a diagnostic benchmark [7, 15]. Among them, seven cases of tMZL were selected for the whole-exome sequencing study. Cases were diagnosed and treated at the Affiliated Hospital of Qingdao University, Qingdao, China, between 2012 and 2021. All cases were reviewed by hematopathologists and diagnosed according to the International Consensus Classification (ICC) 2022 [16].

Clinical data were obtained by reviewing the patients’ medical records. The use of materials and clinical information was approved by the research ethics committee of the Affiliated Hospital of Qingdao University and conformed to the Declaration of Helsinki (no. 943311920, 2021-02-24). Written informed consent was obtained from each patient or a family member.

### Immunohistochemistry

Immunohistochemistry was performed on formalin-fixed, paraffin-embedded tissue sections, antibodies against the following antigens were used for the diagnosis: CD10 (UMAB235, ZSGB-Bio), Bcl-2 (SP66, Roche), Bcl-6 (LN22, ZSGB-Bio), MUM1 (EP190, ZSGB-Bio), MYC (EP121, ZSGB-Bio), CD5 (UMAB9, ZSGB-Bio), and Ki67 index (30-9, Roche). *In situ* hybridization (ISH) with an EBER1 probe was performed to detect Epstein-Barr virus (EBV) infection. The positive threshold was defined as 40% for MYC, 70% for Bcl-2 and 30% for Bcl-6.

### Comparison of clinicopathological characteristics

The progression-free survival (PFS) and overall survival (OS) after HT were determined using the Kaplan-Meier estimator, followed by the Log-rank test to assess the statistical significance of differences between groups’ survival rates. The prognostic factors included patient sex, age, stage, international prognostic index (IPI) score, type of transformed lymphoma, HT in the lymph node site and other sites, treatment for HT, and synchronous versus asynchronous HT (Synchronous HT in lymphoma refers to the simultaneous diagnosis of an indolent and its aggressive transformed state, while asynchronous HT describes the progression of an indolent lymphoma to an aggressive form at a later time point). In this study, *P* values < 0.05 indicated statistical significance.

### Whole-exome sequencing of patients with tMZL

Tumour cell DNA was extracted from formalin-fixed paraffin-embedded (FFPE) samples, and peripheral blood served as a germline control. Genomic DNA from seven patients with tMZL was used to construct sequencing libraries using Agilent SureSelect Human All Exon v6 Kit (Agilent Technologies) according to the manufacturer’s protocol. In these seven samples, tMZL33 (patient case 33) included a biopsy of transformed MZL from two different time points, while tMZL35 contained transformed and non-transformed biopsies. High-throughput paired-end sequencing was performed on the Illumina HiSeq 2500 system (Illumina, San Diego, CA, USA) by Novogene Bioinformatics Technology Co. Ltd (Beijing, China).

### Sequencing data analysis

The paired-end raw reads (Illumina) from each library were mapped to the reference human genome hg38/NCBI GRCh 38 using BWA v 0.7.17 [17], local realignment and deduplication were performed using Genome Analysis Toolkit (GATK) v4.1.2.0 [18]. Somatic mutations were detected using Varscan2 and MuTect v2 [19]. Variant filtration was performed using NCI’s Genomic Data Commons (GDC) workflows. Variants were annotated based on catalogue of somatic mutations in cancer (COSMIC), the 1000 Genomes Project, and dbSNP138 using Oncotator [20]. Variants with allele frequencies that were >0.1% were excluded. To improve variant detection accuracy in FFPE samples, our optimized analysis includes: (1) Filtering unmapped reads; (2) Removing duplicate reads; (3) Adjusting MuTect software parameters for mutation detection, like enhancing alignment quality and modifying strand bias settings; (4) Implementing stringent strand bias filters for C > T mutations. Sanger sequencing was performed to validate somatic mutations in the NF-κB pathway, including mutations in the *TNFAIP3, BCL10* and *CD79B* genes. DNA samples were amplified using standard conditions with primers, each sample was amplified and sequenced using the Sangon Biotech Sanger sequencing platform.

To estimate somatic copy number alterations (CNA) for the tMZL samples, GATK v4.1.2.0 was used to calculate relative coverage depth. CNA regions were detected using the hidden Markov model (HMM) algorithm, and the GISTIC2.0 [21] program was used to identify genes affected by the CNA across all tMZL samples.

## Results

### Patient characteristics in three common types of transformed lymphoma

A total of 35 patients with biopsy confirmed transformed lymphoma were identified among 306 MZL, 544 FL and 871 CLL/SLL cases, the median (range) age of the whole series was 61 (4–95) years and 45.7% were women. Clinical information of patients with transformed lymphoma is summarized in Tables 1 and Supplementary Table S1. All patients with transformed lymphoma developed HT to DLBCL. Seventeen of the 35 transformed lymphomas were tMZL, 11 were tFL, and 7 were tCLL/SLL. Sixteen (45.7%) patients were women, and the median age was 59 years (range, 26–79 years). The median time to HT was 35 months. The median time between MZL and tMZL diagnosis was 26.5 months, the median time between FL and tFL diagnosis was 41.5 months, whereas the time between CLL/SLL and tCLL/SLL diagnosis was 23 months. Lymph nodes were the major sites of HT (80%). Treatment strategies varied according to the stage, age, tumour size and type of transformed lymphoma. The 5-year OS and PFS rates after HT were 50% and 26%, respectively. A total of 15 patients died of the transformed lymphoma.

**Table 1.** Characteristics of the patients with transformed lymphoma.

Parameters	tFL (*n* = 11)		tMZL (*n* = 17)		tCLL/SLL (*n* = 7)	
	Synchronous	Asynchronous	Synchronous	Asynchronous	Synchronous	Asynchronous
Number of cases	7	4	13	4	6	1
Age, median (range)^#^	61 y (46–74)	58.5 y (46–71)	58 y (29–75)	65 y (49–68)	64 y (26–79)	56 y
Sex, male (%)	4 (57%)	2 (50%)	7 (54%)	1 (25%)	4 (67%)	1 (100%)
Stage, III-IV (%)^#^	5 (71%)	4 (100%)	11 (85%)	4 (100%)	4 (67%)	1(100%)
IPI, ≥3 (%)^#^	3 (43%)	1 (25%)	6 (46%)	3 (75%)	2 (33%)	0
Time to HT	NA	41.5 m (17-48)	NA	26.5 m (14-53)	NA	23 m
Site of HT						
Lymph node	7	3	10	3	5	
Other sites		1	3	1	1	1
Treatment for HT						
R-CHOP	1	2	7	1	1	1
CHOP	2	2	1		1	
R-CDOP	4		4	1	1	
Other		1	1	2	1	
Not available					2	
Response to treatment						
CR	5	1	6	1	1	
PR	2	1	4	1	1	
SD/PD		2	3	1	2	1
Not available				1	2	

^#^Data for age, stage, and IPI are at HT.

FL: follicular lymphoma; MZL: marginal zone lymphoma; CLL/SLL: chronic lymphocytic leukaemia/small lymphocytic lymphoma; DLBCL: diffuse large B-cell lymphoma; HT: Histological transformation; NA: not available; R: rituximab; CHOP: cyclophosphamide, doxorubicin, vincristine, and prednisone; CDOP: cyclophosphamide, liposomal doxorubicin, vincristine, and prednisone; CR: complete response; PR: partial response; SD: stable disease; PD: progressive disease.

### Immunohistochemical results of transformed lymphoma

**Figure 1.**
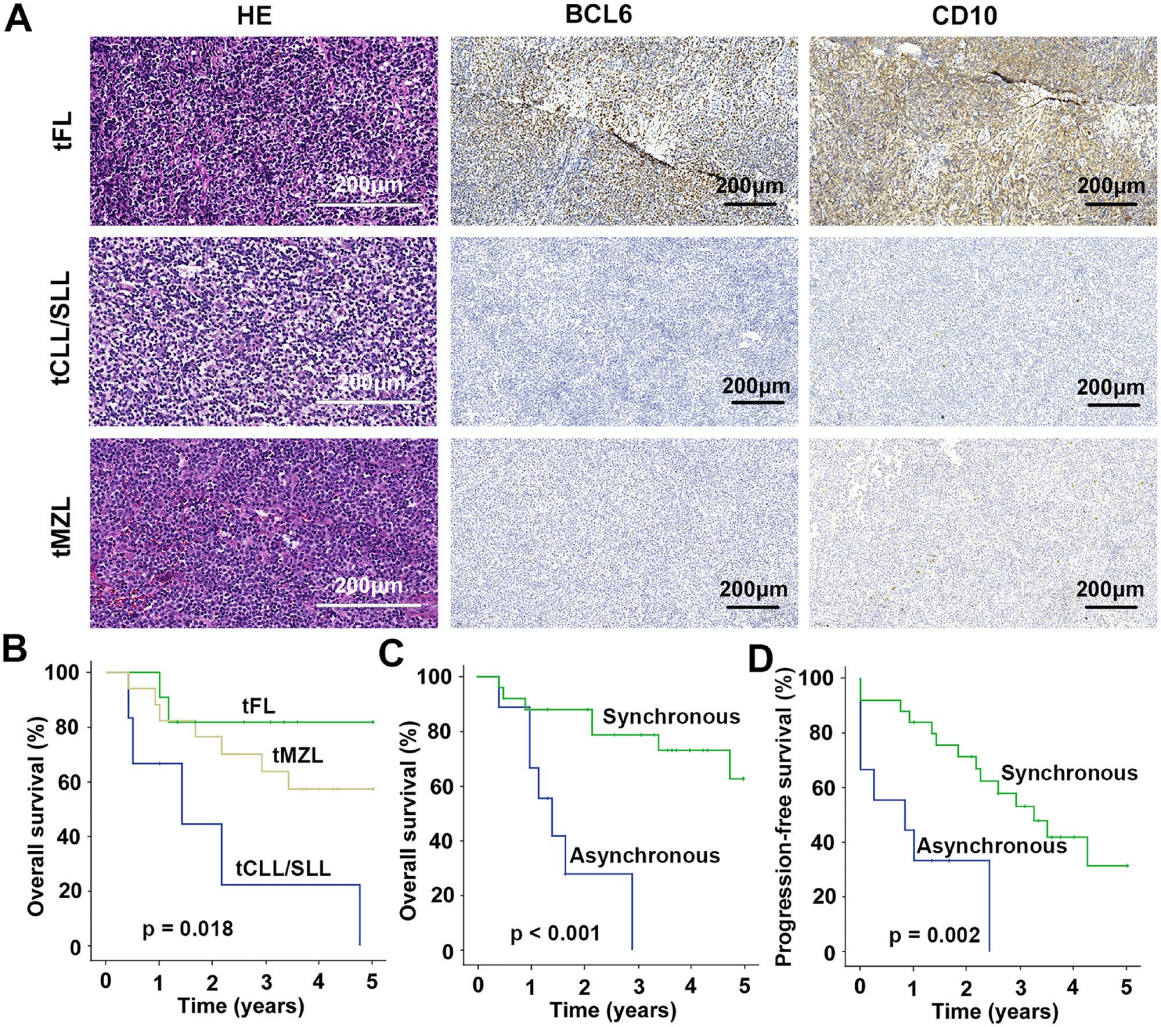
Clinicopathological characteristics of transformed lymphoma. (A) Histological characteristics of transformed lymphoma. Haematoxylin and eosin (HE) (×400), BCL6^+^ (×200) and CD10^+^ (×200) results of tFL, tCLL/SLL and tMZL. (B) OS in tFL, tMZL and tCLL/SLL. (C) OS in synchronous and asynchronous HT patients. (D) PFS in synchronous and asynchronous HT patients. OS, overall survival; PFS, progression-free survival; HT, histological transformation; tFL, transformed follicular lymphoma; tMZL, transformed marginal zone lymphoma; tCLL/SLL, transformed chronic lymphocytic leukaemia/small lymphocytic lymphoma

The immunohistochemical findings of the 35 transformed lymphomas are summarized in Table 2. GCB-type was observed in 23% (7/31) of the transformed lymphomas cases, while the remaining 77% (24/31) were non-GCB. Immunohistochemical results of the transformed lymphomas were as follows: CD10, 24% (8/33); Bcl-6, 50% (17/34); MUM1, 65% (20/31); MYC, 30% (6/20); Bcl-2, 79% (27/34); CD5, 32% (9/28); and Ki67 ≥ 60%, 53% (18/34). The majority of tFL expressed CD10 (64%) and Bcl-6 (91%), while their expression was lower in tMZL (CD10, 6%; Bcl-6, 29%) and tCLL/SLL (CD10, 0%; Bcl-6, 33%) (Figure 1(A)).

**Table 2.** Histopathological and immunohistochemical results of transformed lymphoma.

Histopathology of transformation	Patients	GCB (%)/ non-GCB (%)	CD10 (%)	Bcl-6 (%)	MUM1 (%)	MYC (%)	Bcl-2 (%)	CD5 (%)	Ki67 ≥ 60% (%)
tFL	11	6 (55)/5 (45)	7 (64)	10 (91)	9 (82)	3 (43)	7 (64)	1 (13)	6 (60)
						(4 NA)		(3 NA)	(1 NA)
tMZL	17	1 (7)/14 (93)	1 (6)	5 (29)	8 (53)	3 (27)	13 (81)	3 (23)	9 (53)
		(2 NA)			(2 NA)	(6 NA)	(1 NA)	(4 NA)	
tCLL/SLL	7	0 /5 (100)	0	2 (33)	3 (60)	0	7 (100)	5 (71)	3 (43)
		(2 NA)	(2 NA)	(1 NA)	(2 NA)	(5 NA)			
Total	35	7 (23)/24 (77)	8 (24)	17 (50)	20 (65)	6 (30)	27 (79)	9 (32)	18 (53)
		(4 NA)	(2 NA)	(1 NA)	(4 NA)	(15 NA)	(1 NA)	(7 NA)	(1 NA)

NA, not available.

### Survival analysis in transformed lymphoma

Among the 35 patients with transformed lymphoma included in this study, 34 were successfully followed up, while one patient was not followed up. The last follow-up date was 6 September 2021. The median follow-up time was 48.5 months. During the follow-up period, 15 patients died of transformed lymphoma. The outcome details of these patients are presented in Table S1

Additionally, the Kaplan–Meier curves of OS and PFS for transformed lymphoma are shown in Figure 1(B–D). Kaplan–Meier survival analyses of patients stratified by sex, age, stage, IPI score, type of transformed lymphoma, HT in the lymph node site and other sites, treatment for HT and synchronous versus asynchronous HT were also performed. The results indicated that tCLL/SLL and asynchronous HT (indolent lymphoma followed by DLBCL) were significantly associated with an inferior OS, while asynchronous HT was significantly associated with an inferior PFS (Figure 1(B–D)). The Log-rank test and Cox regression analysis results also indicated significant differences in OS based on the type of transformed lymphoma and whether synchronously or asynchronously HT, with both factors showing statistical significance (*p* < 0.05) (Figure S1).

### The clinicopathological characteristics, somatic mutations and CNA profiles of tMZL

Among the 35 consecutive patients with transformed lymphoma, tMZL was observed in 17 cases. The median age was 58 years (range, 29–75 years). Thirteen patients (76%) had synchronous MZL and DLBCL HT (Table 1), and the remaining four patients (24%) developed MZL with subsequent DLBCL HT. Immunohistochemical results of the tMZL were as follows: CD10, 6% (1/17); Bcl-6, 29% (5/17); MUM1, 53% (8/15); MYC, 27% (3/11); Bcl-2, 81% (13/16); CD5, 23% (3/13); and Ki67 ≥ 60%, 53% (9/17). GCB-type was observed in 7% (1/15) of the cases, while the remaining 93% (14/15) were non-GCB (Table 2). The Kaplan–Meier curves of OS for tMZL are shown in Figure S2, asynchronous MALT lymphoma and DLBCL (*p* = 0.001) was a significant adverse factor for OS. The prognosis of patients with tMZL and tFL was not significantly different.

**Figure 2.**
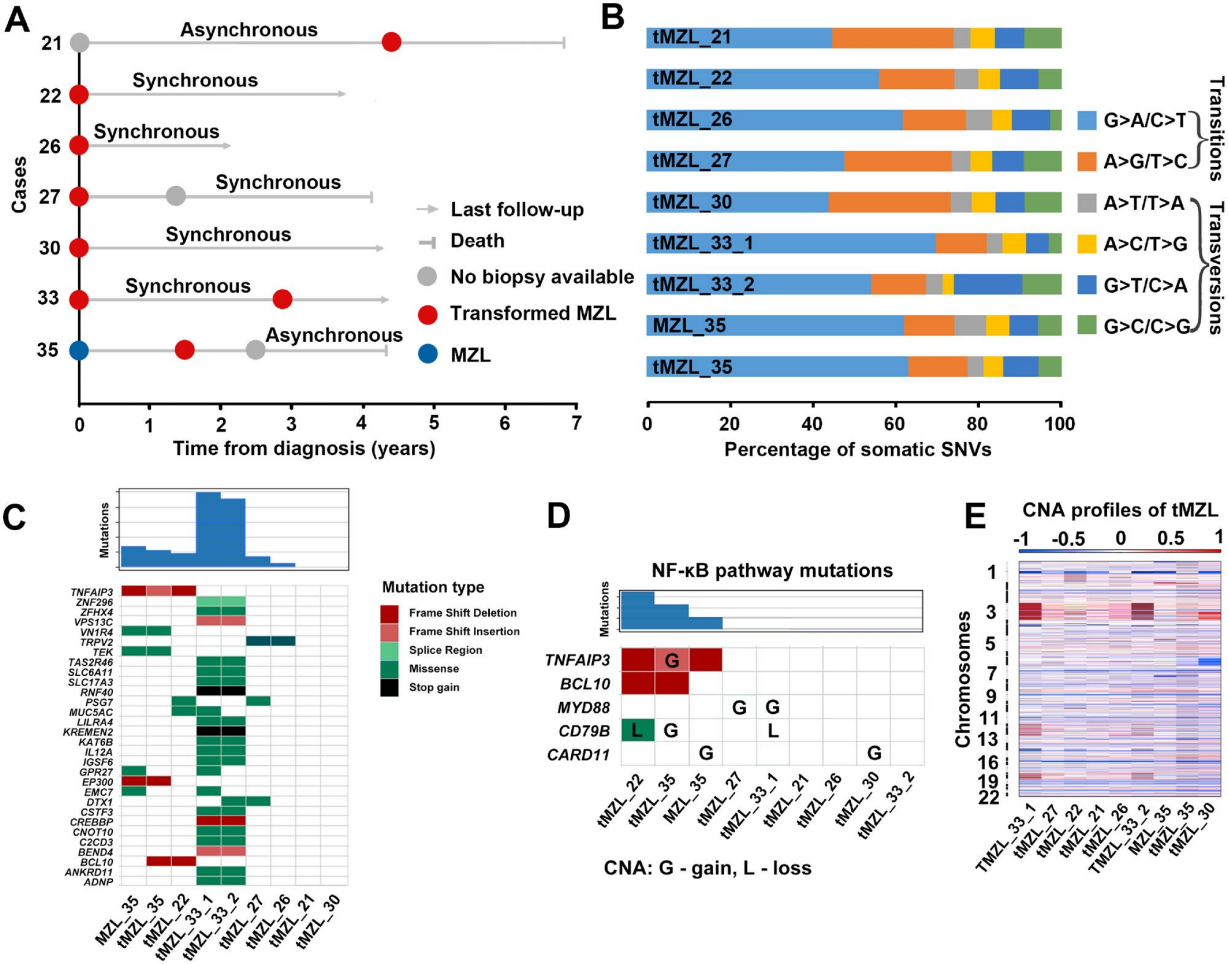
Clinical timelines and mutation profiles for tMZL cases. (A) Disease event timeline and biopsy information of 7 whole-exome sequenced tMZL cases. (B) Distribution of base substitution patterns for all somatic mutations. (C) The landscape of recurrently mutated genes in tMZL. The top cell indicates the number of mutations detected in our cases. (D) Somatic variants in NF-κB signalling pathway genes. (E) Copy number alterations heatmap in tMZL cases. MZL, marginal zone lymphoma; tMZL, transformed marginal zone lymphoma

We achieved an average sequencing depth of 231× (range 172×–297×) for tumour tissues and 158× (range 113×–209×) for germline controls. In the seven whole-exome sequenced tMZL cases, we detected 217 non-synonymous variants affecting 174 genes (Figure 2(A); Table S2). The most common nucleotide substitutions were G > A/C > T transitions (Figure 2(B)).

A total of 30 genes were recurrently mutated in the tMZL samples (19 of these were found in 2 biopsies from tMZL33) (Figure 2(C)), CNAs in tMZL were also identified (Figure 2(E), Figure S3, Table S3). Our study found that *TNFAIP3* was one of the most frequently mutated gene in tMZL samples (tMZL22, tMZL35) (Figure S4), which has been previously reported to be frequently mutated in lymphomas [22, 23]. Examination of alterations in key pathways implicated in B-cell lymphomas [24, 25] suggests involvement in chromatin remodelling (*CREBBP* and *EP300*) and regulators of NF-κB signalling (*TNFAIP3, BCL10, MYD88, CD79B* and *CARD11*) (Figure 2(D)) were affected.

### Patterns of evolution from MZL to tMZL

**Figure 3.**
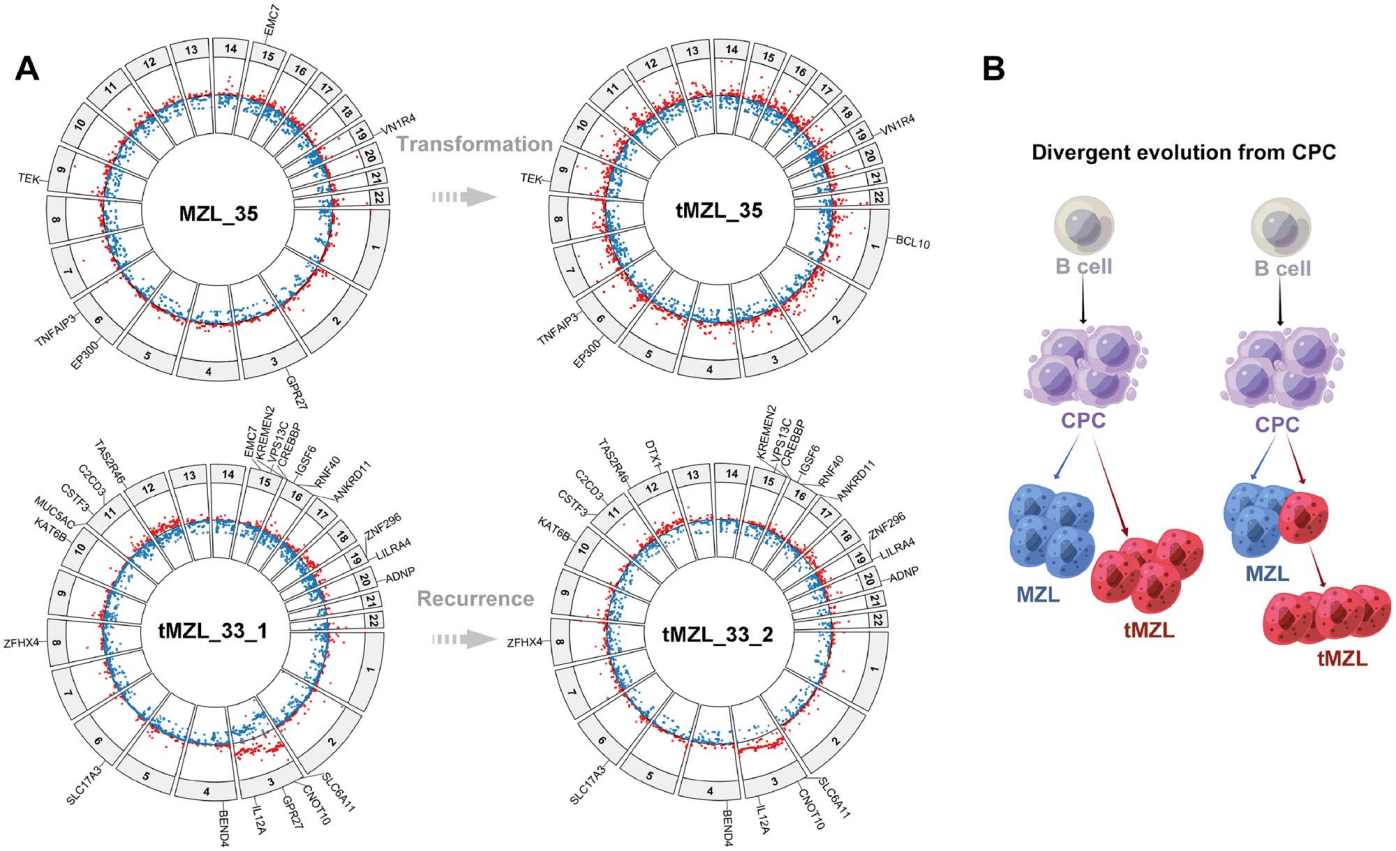
Inferred model of clonal evolution during MZL transformation. (A) Recurrent somatic mutations and copy number alterations in patient 33 and 35. The outer ring of the plot shows the chromosomes and recurrent mutations, the inside ring shows copy number gains (red) and losses (blue). (B) Evolution of tMZL. MZL and tMZL arise through divergent evolution, in the divergent model, the MZL and tMZL dominant clones derive from an ancestral common progenitor clone (CPC) through the independent acquisition of distinct mutations. Two different schematics in Figure 3(B) represent asynchronous and synchronous HT. MZL, marginal zone lymphoma; tMZL, transformed marginal zone lymphoma

The divergent evolutionary pattern was explained by patient 35 (Figure 3), who had asynchronous HT, and was studied at the time of two events. Some of the mutations were preserved during transformation as her disease progressed. Meanwhile, many novel mutations appeared in the process of HT. Moreover, we also found that some mutations appearing at MZL disappeared or became undetectable in the tMZL. This would mean that MZL and tMZL arise from a commonly mutated progenitor through the independent acquisition of different genetic mutations. This divergent evolutionary pattern was also applied to patient 33, who had a recurrence of tMZL. Furthermore, patient 33 had a decreased tumor mutation burden after recurrence.

## Discussion

Here, we retrospectively analysed the immunophenotypes, prognostic factors and patient outcomes of transformed lymphoma. To further evaluate the genetic basis of HT and to find genomic alterations and key pathways that could be exploited therapeutically, we performed a whole-exome sequencing study of seven tMZL cases.

In our study, the 5-year OS rate of patients with DLBCL HT of FL was 79% compared to 46% to 87% reported in previous studies [6, 26]. The 5-year OS rate of patients with tMZL has been reported to be 33%–94% [7, 8, 27, 28]. In the present study, the 5-year OS rate of tMZL was 57%. In the current study, the median OS of patients with tCLL/SLL was 22.5 months, which was longer than the OS reported by Elnair et al. [29,  30]. This might be partly due to the young age and low IPI scores of most of the patients included in the present study.

Immunohistochemical results for Bcl-6 and CD10 were positive in 91% and 64% of patients, respectively, among those with tFL. Bcl-6 and CD10 demonstrated lower expression in tMZL (Bcl-6, 29%; CD10, 6%) and tCLL/SLL (Bcl-6, 33%; CD10, 0%). This indicates that although these three indolent lymphomas were transformed into DLBCL, their immunophenotypes are still different.

Prognostic factors for the comparison of different types of transformed lymphoma have been less previously reported [6, 31, 32]. Our data show that the OS of tFL was better than that of tMZL, while tCLL/SLL had the poorest OS. Synchronous versus asynchronous HT (*p* < 0.001), and type of transformed lymphoma (*p* = 0.018) were significant prognostic factors for OS. Asynchronous HT (*p* = 0.002) was a significant adverse factor for PFS. Previous studies have reported that patients with synchronous transformed lymphoma had a better OS and PFS than those with asynchronous transformed lymphoma [7, 33, 34], which is consistent with the results of the present study. The current study also showed that advanced-stage disease at HT was not an adverse prognostic factor for PFS or OS, which may be related to the fact that most cases of transformed lymphoma are at an advanced stage. A previous study reported that the survival of patients with transformed lymphoma improved in the rituximab era [35]. However, HT treatment was not a significant adverse prognostic factor for OS and PFS in the present study. Because the majority of the transformed lymphomas were treated with R-CHOP, only seven patients with transformed lymphoma did not receive rituximab.

The present study revealed extensive genetic heterogeneity, there is a large number of genetic variations affecting many genes and pathways, reflecting the complexity of the HT process. Our results suggest that the genomic mutational landscape and CNA profiles of tMZL is much more complex than that of MZL [22, 36, 37]. We identified chromatin regulator genes were mutated in MZL (*EP300*) and tMZL (*CREBBP* and *EP300*), previous studies demonstrated that chromatin regulator genes mutations were early events in MZL [36, 38]. Somatic mutations within regions of CNAs detected in our study, such as those in *TNFAIP3, BCL10,* and *CD79B* are associated with NF-κB activation and frequently mutated in de novo ABC-DLBCL [24, 39]. Therefore, it is likely that they also have similar functional roles in tMZL. Previous studies strongly suggest that activation of the NF-κB pathway is closely associated with the transformation of Waldenström macroglobulinemia and FL [11, 40], it may have also played an important role in the transformation of MZL. Many therapeutic agents target the NF-κB signalling pathway, such treatment might be beneficial for MZL patients harbouring mutations in this pathway. In addition, two patients (tMZL21 and tMZL30) did not present somatic mutations in any of these genes. But we found particular CNAs at tMZL30, such as amplifications of *CARD11* which affecting regulators of NF-κB signalling at transformation (Figure 2(D)) [11]. We identified non-synonymous mutations in *KIR3DL3* at tMZL21 (Table S2), which played an important role during cancer development [41].

Consistent with the divergent evolution model, our analysis corroborates the dominant tMZL clone arises from common progenitor clone (CPC) through the acquisition of independent genetic events. The existence of CPC can be postulated based on the presence of a common set of lesions between MZL and tMZL, which is consistent with previous studies based on FL progression [42]. This divergent evolutionary pattern was also applied to recurrence of tMZL. The limitation of this study is the small sample size, especially the likelihood that the small sequenced cohort does not adequately represent the breadth/heterogeneity of mutations in tMZL.

In conclusion, the 5-year OS and PFS rates after DLBCL HT were 50% and 26%, respectively. The OS and PFS of patients with tFL were higher than those of tCLL/SLL and tMZL. Furthermore, Bcl-6 and CD10 were positive in 91% and 64% of tFL, respectively, while Bcl-6 and CD10 were less expressed in tMZL and tCLL/SLL. This indicates that although these three indolent lymphomas were transformed into DLBCL, their immunophenotypes are still different. Kaplan-Meier survival analysis revealed that asynchronous HT and tCLL/SLL were significant adverse prognostic factors for OS after DLBCL HT. In terms of the mutation landscape of tMZL, although this is only the first step, it seems likely that certain alterations contribute to the occurrence of aggressive disease, particularly the activation of the NF-κB pathway. More research is needed to better understand the genetic mechanisms underlying HT, this knowledge will help identify promising therapeutic targets in the future. Accordingly, the results got from this study will be the first step for further investigation using large cohort.

## Supplementary Material

Supplemental Material

## Data Availability

The datasets obtained from web-based sources and subsequently analysed in our study were: human genome (hg38) (http://hgdownload.soe.ucsc.edu/goldenPath/hg38/bigZips/), dbSNP Build 138 (https://www.ncbi.nlm.nih.gov/snp/), Mills & 1000 G Gold Standard Indels (GATK resource bundle) (ftp://ftp.broadinstitute.org/bundle/hg38/), and ClinVar database vcf_GRCh38 (https://ftp.ncbi.nlm.nih.gov/pub/clinvar/vcf_GRCh38/). The other data sources utilized in this study are all subject to local, ethical and privacy restrictions for data transfer abroad or into public domain limiting data availability on request. The dataset used and/or analyzed during the current study are available from the corresponding author on reasonable request.
