# Supplementary material for: Clinicopathological characteristics and genomic profiling in patients with transformed lymphoma: a monocentric retrospective study
Source: Ann Med. 2024 Oct 26;56(1):2419556. doi: 10.1080/07853890.2024.2419556 (PMC11514389; doi:10.1080/07853890.2024.2419556)
Supplement: Supplemental Material [file IANN_A_2419556_SM1380.zip › suppl_data/Suppl_Figure caption.docx]

**Supplementary Material**

Figure S1 Cox regression analysis of OS associated clinical features

Figure S2 prognostic factors in patients with tMZL

Figure S3 Recurrent somatic mutations and copy number alterations in tMZL cases

Figure S4 *TNFAIP3* mutations in tMZL
