# Supplementary figures and images for: Clinicopathological characteristics and genomic profiling in patients with transformed lymphoma: a monocentric retrospective study
Source: Ann Med. 2024 Oct 26;56(1):2419556. doi: 10.1080/07853890.2024.2419556 (PMC11514389; doi:10.1080/07853890.2024.2419556)

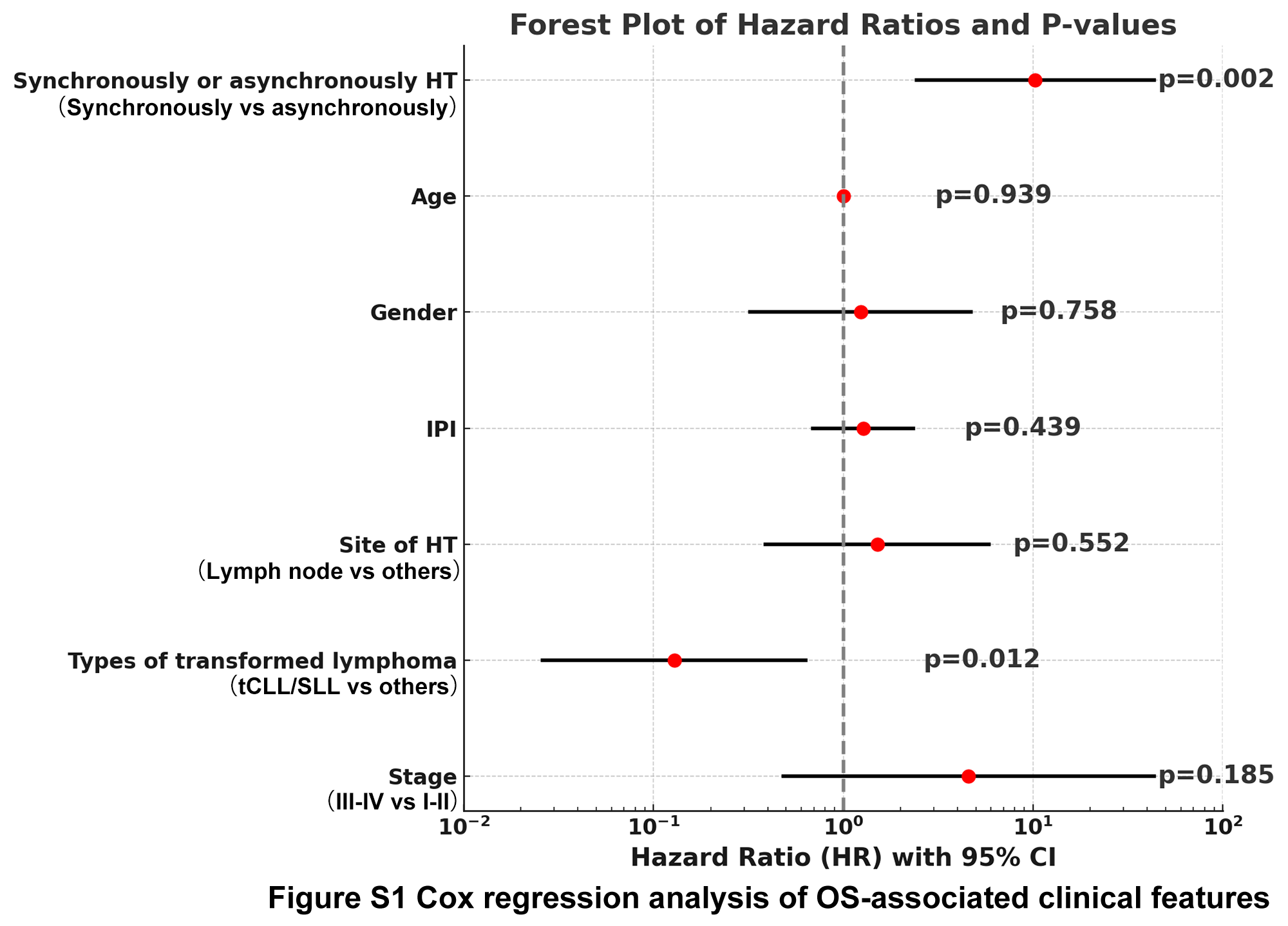

Supplement: Supplemental Material [file IANN_A_2419556_SM1380.zip › suppl_data/Figure_S1_Cox_regression_analysis_of_OS_associated_clinical_features.tif]

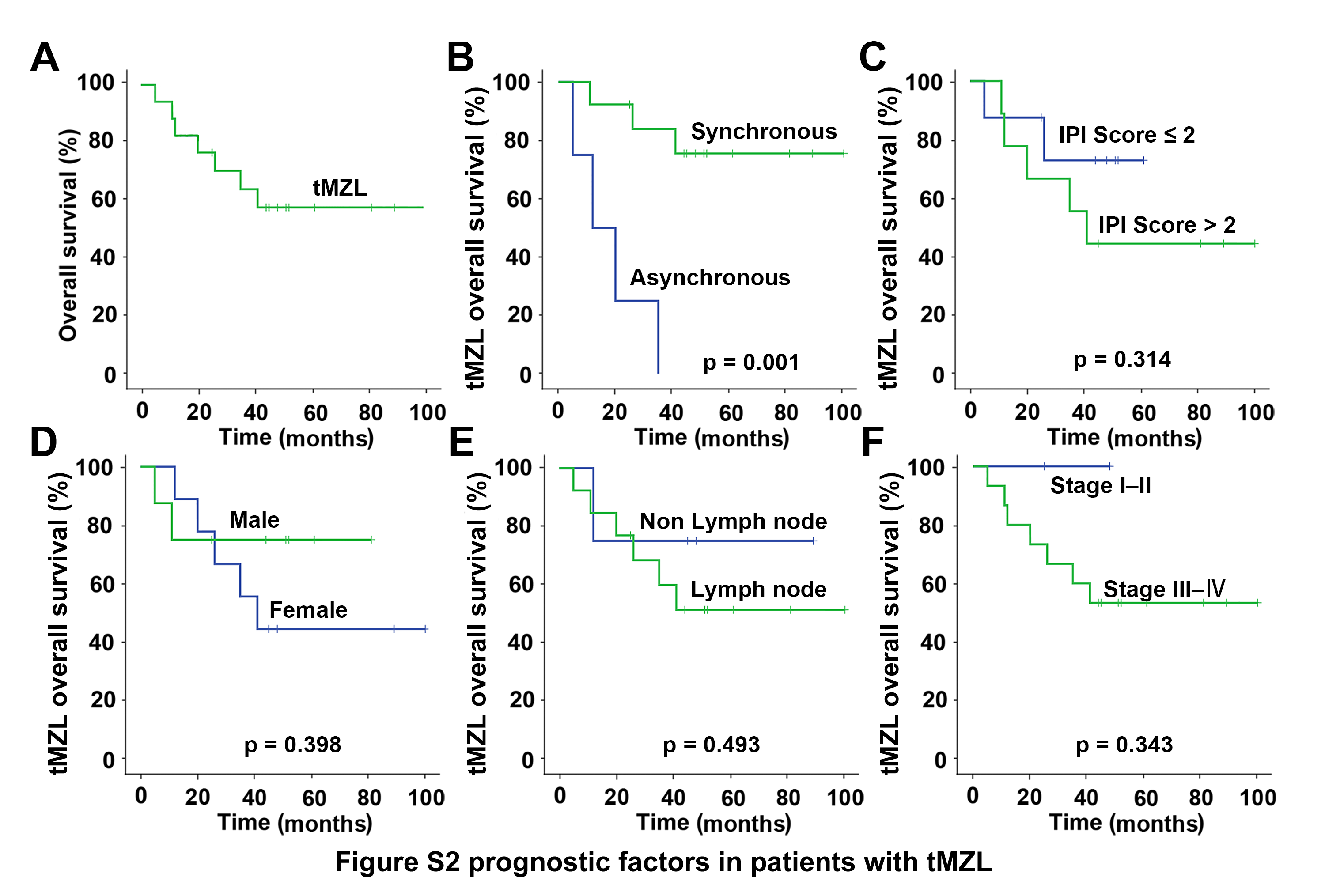

Supplement: Supplemental Material [file IANN_A_2419556_SM1380.zip › suppl_data/Figure_S2_prognostic_factors_in_patients_with_tMZL.tif]

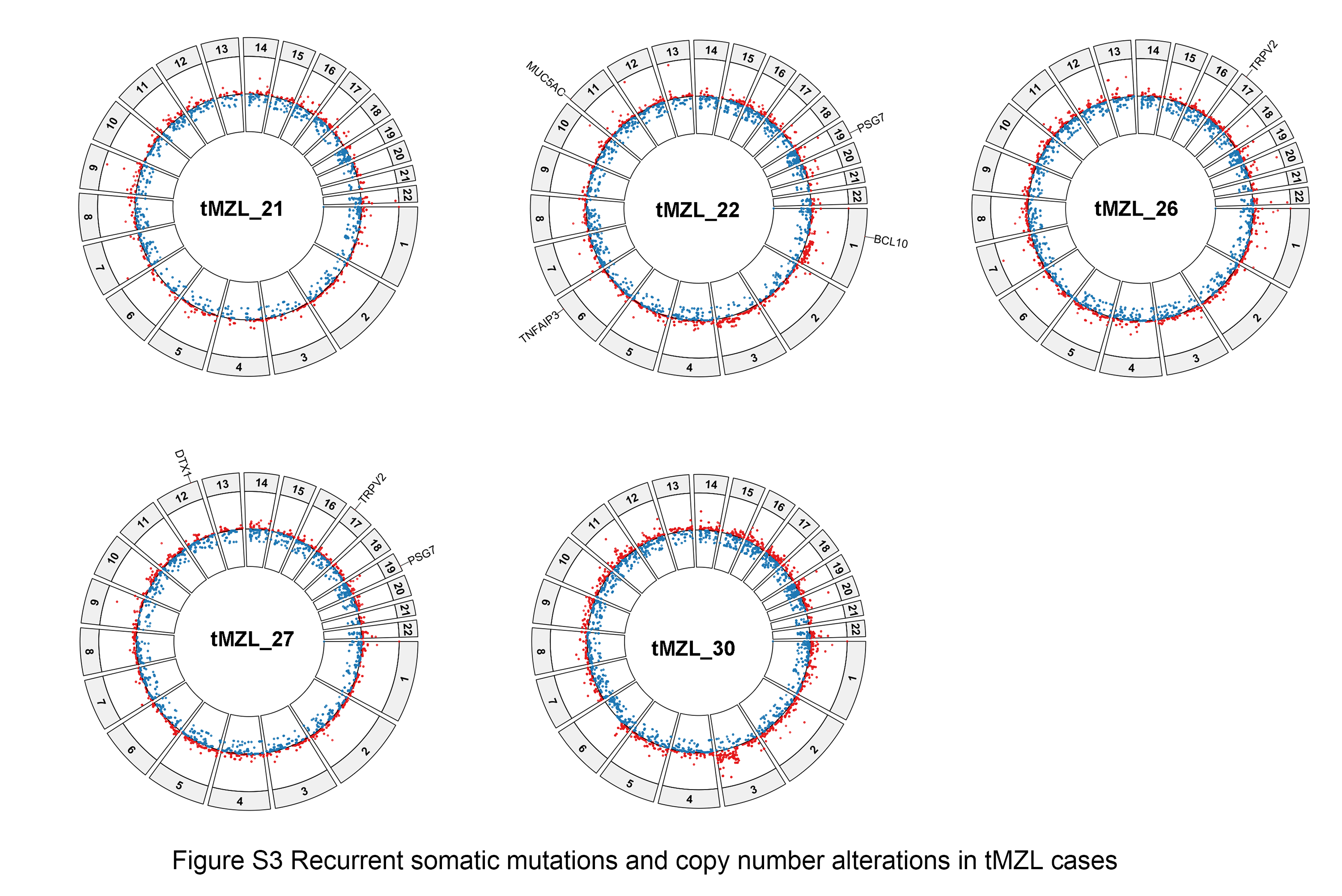

Supplement: Supplemental Material [file IANN_A_2419556_SM1380.zip › suppl_data/Figure_S3_Recurrent_somatic_mutations_and_copy_number_alterations_in_tMZL_cases.tif]

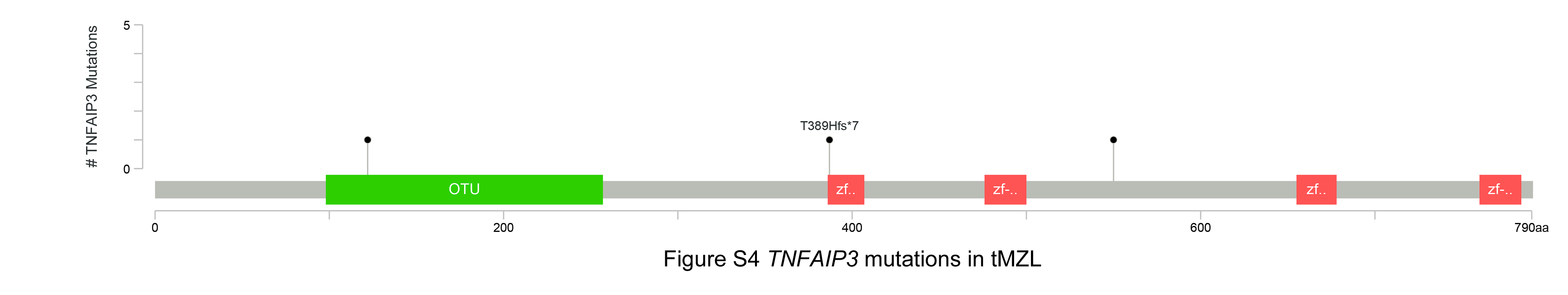

Supplement: Supplemental Material [file IANN_A_2419556_SM1380.zip › suppl_data/Figure_S4_TNFAIP3_mutations_in_tMZL.tif]

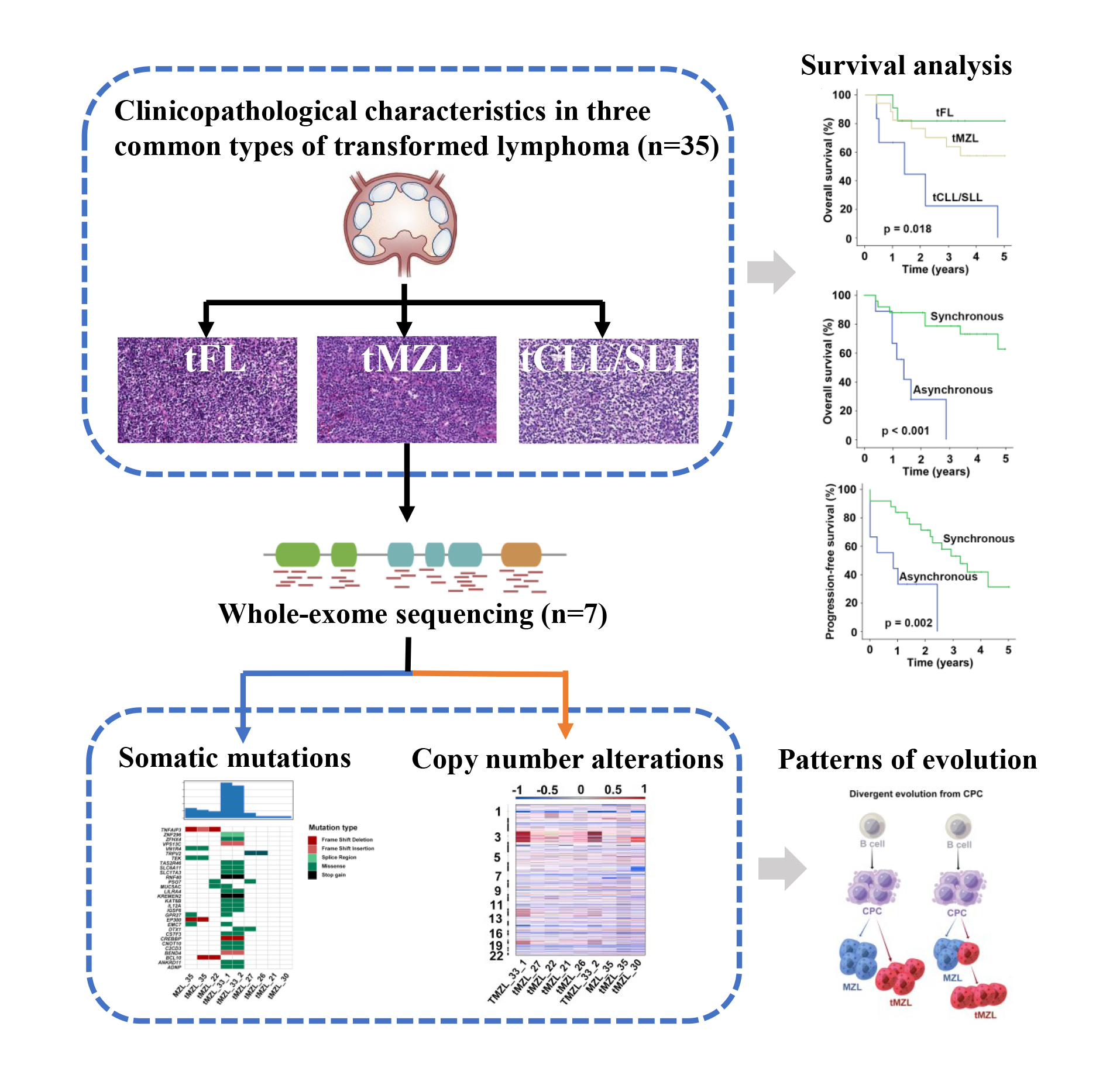

Supplement: Supplemental Material [file IANN_A_2419556_SM1380.zip › suppl_data/Graphical abstract.tif]
